# Alcohol Intake and Binge Drinking and Their Association With Chronic Obstructive Pulmonary Disease, Emphysema, and Chronic Bronchitis: A Retrospective Study Using the Behavioral Risk Factor Surveillance System (BRFSS) Database

**DOI:** 10.7759/cureus.90371

**Published:** 2025-08-18

**Authors:** Harikaran Reddy Ragireddy, Erica Lama, Mehal Ravindra Adsure, Aditya Rajesh Pawar, Juveria Fatima, Samuel Devasish

**Affiliations:** 1 Internal Medicine, Sriramachandra Institute of Higher Education and Research, Chennai, IND; 2 Internal Medicine, Manipal Teaching Hospital, Pokhara, NPL; 3 Internal Medicine, Shrimati Kashibai Navale Medical College and General Hospital, Pune, IND; 4 Internal Medicine, Deccan College of Medical Sciences, Hyderabad, IND; 5 Internal Medicine, Andhra Medical College, Visakhapatnam, IND

**Keywords:** alcohol intake, binge drinking, brfss, chronic bronchitis, chronic obstructive pulmonary disease, copd, emphysema, odds ratio, retrospective study

## Abstract

Background and objective

While alcohol intake and binge drinking have been associated with various types of cancers, their relationship with chronic obstructive pulmonary disease (COPD) remains unclear. Understanding this association is crucial to identifying potential behavioral or biological factors influencing COPD, emphysema, or chronic bronchitis risk. This study aimed to evaluate the association between binge drinking and COPD, emphysema, or chronic bronchitis, and assess the odds of developing COPD in participants with binge drinking based on demographic and socioeconomic characteristics.

Methodology

This retrospective study utilized the 2022 Behavioral Risk Factor Surveillance System (BRFSS) database. The disease variables were self-reported chronic COPD, emphysema, or chronic bronchitis, and the risk factor was binge drinking (Yes/No). Control variables included demographic (age, gender, race) and socioeconomic characteristics (education, annual income). Data were analyzed using cross-tabulations, chi-square tests, and Fisher’s exact tests, with results expressed as odds ratios (ORs) and 95% confidence intervals (CIs).

Results

Participants with a history of binge drinking had a 37.4% lower likelihood of developing COPD, emphysema, or chronic bronchitis (OR: 0.626, 95% CI: 0.602-0.65). There was also a statistically significant lower likelihood in the 25-44 years age group (13.3% lower likelihood, OR: 0.867, 95% CI: 0.789-0.952). Female participants showed a greater reduction (38.6% lower likelihood, OR: 0.614, 95% CI: 0.579-0.65) when compared to male participants (32.6% lower likelihood, OR: 0.674, 95% CI: 0.64-0.71). Racial disparities indicated that white, non-Hispanic participants had the most significant reduction in COPD risk (40.1% lower likelihood, OR: 0.599, 95% CI: 0.573-0.625). Socioeconomic factors revealed that participants with advanced education had a 40.5% lower likelihood (OR: 0.595, 95% CI: 0.565-0.626), while participants with lower (OR: 0.7, 95% CI: 0.662-0.74) and higher annual incomes (OR: 0.7, 95% CI: 0.656-0.746) both had a 30% lower likelihood.

Conclusions

Based on our findings, binge drinking is inversely associated with COPD, emphysema, or chronic bronchitis risk across demographic and socioeconomic groups. Further prospective studies are needed to confirm this association and explore the underlying mechanisms.

## Introduction

Chronic obstructive pulmonary disease (COPD) is a progressive lung disease characterized by persistent respiratory symptoms and airflow limitation. It includes conditions like emphysema and chronic bronchitis. COPD is primarily caused by smoking, air pollution, and genetic factors, and it poses a significant public health challenge globally. In 2019, COPD affected an estimated 212 million people worldwide, making it the third leading cause of death [1]. In the United States alone, over 16 million adults were diagnosed with COPD in 2020, though many more may be undiagnosed [2]. COPD significantly reduces the quality of life due to symptoms such as chronic cough, shortness of breath, and excessive mucus production. These symptoms worsen over time, leading to reduced physical capacity and chronic fatigue. Breathlessness in particular limits daily activities, contributing to social isolation and diminished mental health [3]. As COPD progresses, patients often require oxygen therapy and other interventions to manage symptoms.

Although alcohol consumption is not a direct cause of COPD, it can exacerbate the disease through its impact on lung function and the immune response. Excessive alcohol intake weakens the body's antioxidant defenses, leading to oxidative stress that damages lung tissue. This damage is compounded by alcohol’s impairment of the cilia, which play a vital role in clearing mucus and pathogens from the lungs. This dysfunction can lead to recurrent respiratory infections, a key factor in COPD progression [4].

A study published in 2016 found that heavy alcohol use is associated with an increased risk of respiratory infections in individuals with COPD, further worsening their lung function [4]. Another study published in 2020 showed that alcohol abuse increases oxidative stress and inflammation, both of which contribute to the progression of COPD [5]. Although the exact mechanisms by which alcohol influences COPD are not fully understood, it is clear that the immune system is weakened by excessive alcohol intake, increasing the likelihood of infections and exacerbations. In addition to weakening the immune response, alcohol disrupts the balance of antioxidants in the lungs, further impairing the body's ability to manage oxidative stress. This not only damages lung tissue but also hampers the body's natural defenses against pollutants and irritants, leading to inflammation and an increased risk of respiratory illnesses [6].

The Behavioral Risk Factor Surveillance System (BRFSS) is a nationwide telephonic survey conducted annually in the US. It collects self-reported data on health-related behaviors, chronic health conditions, and preventive practices among adults. The BRFSS is one of the largest population-based tools for monitoring health trends and identifying potential associations between lifestyle factors and diseases. In this study, we aim to evaluate the association between alcohol intake and COPD by using the 2022 BRFSS database, exploring how alcohol intake among adults may influence COPD prevalence. The aims and objectives of this study are to evaluate the association between binge drinking and COPD, emphysema, or chronic bronchitis, and assess the odds of developing COPD in participants with binge drinking and without binge drinking in the age group of 18-65+ years. Participants of both genders and various races (white, non-Hispanic, blank non-Hispanic, and non-Hispanic/other race) were included, along with participants with different education and annual income levels.

## Materials and methods

Study design and setting

An original retrospective research study was carried out using the BRFSS. database [7]. The data for this study were retrieved and analyzed on December 12, 2024. Given that the BRFSS dataset consists of de-identified public data, this study is classified as non-human participant research, and therefore, ethics committee approval was not necessary.

Study population and data collection

Data was extracted using the BRFSS Web-Enabled Analysis Tool (WEAT) for the year 2022. It simplifies the process of examining health-related behaviors, chronic conditions, and risk factors collected through the BRFSS surveys. The tool allows users to customize their analysis by selecting various variables. In this study, the disease variable was COPD, which includes emphysema or chronic bronchitis, based on the response to the question of whether participants were ever told they had COPD, emphysema, or chronic bronchitis (CHCCOPD3). The risk variable focused on binge drinking, defined as men consuming five or more drinks and women consuming four or more drinks on a single occasion (_RFBING6). Control variables included demographic and socioeconomic factors. Demographic factors included age (_AGE_G), classified into four groups (18-24, 25-44, 45-64, and 65+ years); gender (SEX) - categorized as male or female; and race (_RACEGR), with categories for white (non-Hispanic), black (non-Hispanic), and non-Hispanic/other races. Socioeconomic factors included education level (EDUCA), divided into basic education (high school or less) and advanced education (some college or higher); and annual income (_INCOMG1), classified as less than $50,000 or greater than $50,000. Only participants who responded to the queries related to relevant disease, risk factor, and control variables were included in the study to ensure complete data for analysis.

Statistical analysis

Descriptive statistics, including numbers and percentages, were generated for each variable using cross-tabulations in the BRFSS WEAT. The data were exported to Microsoft Excel for organizing and further processing. Statistical analyses were performed using R version 4.3.1. Chi-square tests were used to assess associations between categorical variables, while Fisher's exact test was applied for variables with small sample sizes to ensure accuracy. Results were expressed as odds ratios (ORs) with 95% confidence intervals (CIs). A p-value <0.05 was considered statistically significant.

## Results

In the year 2022, 392,288 people participated in the BRFSS study across the United States. Among them, all the participants answered the relevant questions and were included in the study. Table 1 shows the prevalence of COPD, emphysema, or chronic bronchitis in binge drinkers and non-binge drinkers’ groups. Out of the total 56,705 participants who self-reported as binge drinkers, 3,120 (5.5%) had COPD, emphysema, or chronic bronchitis. In contrast, among the 335,583 participants who did not self-report as binge drinkers, 307,006 (91.5%) did not have COPD, emphysema, or chronic bronchitis. The OR of 0.626 indicates that participants who self-reported as binge drinkers were 37.4% less likely to have COPD, emphysema, or chronic bronchitis compared to non-binge drinkers, and this association is statistically significant.

**Table 1:** Prevalence of binge drinking in participants with self-reported COPD, emphysema, or chronic bronchitis ^*^P-value <0.05 was considered statistically significant CI: confidence Interval; COPD: chronic obstructive pulmonary disease; OR: odds ratio

		COPD, emphysema, or chronic bronchitis, n (%)		OR (CI)
		Yes	No	
Binge drinkers, n (%)	Yes	3,120 (5.5%)	53,585 (94.5%)	0.626 (0.602, 0.65)^*^
	No	28,577 (8.5%)	307,006 (91.5%)	

Table 2 shows the prevalence of COPD, emphysema, or chronic bronchitis in binge drinker and non-binge drinker groups based on demographic characteristics. The prevalence of COPD, emphysema or chronic bronchitis in participants reported as binge drinkers was 99 (1.6%) in the 18-24 years age group, 583 (2.5%) in the 25-44 years age group, 1,373 (7%) in the 45-64 years age group, and 1,065 (13.6%) in participants aged 65 years and above. Regarding gender, COPD, emphysema, or chronic bronchitis was more prevalent in males reported as binge drinkers (n=1,806, 5.2%) compared to females reported as binge drinkers (n=1,314, 5.9%). As for racial groups, white participants reported as binge drinkers had a prevalence of 2,385 (5.7%), Black participants reported as binge drinkers had a prevalence of 225 (6.9%), and non-Hispanic/other race participants reported as binge drinkers had a prevalence of 424 (4%). The likelihood of an association between binge drinkers and COPD, emphysema, or chronic bronchitis, expressed as odds ratios, was consistently lower in binge drinkers compared to non-binge drinkers across all demographic groups.

**Table 2:** Prevalence of binge drinking in participants with self-reported COPD, emphysema or chronic bronchitis based on demographic characteristics ^*^P-value <0.05 was considered statistically significant CI: confidence Interval; COPD: chronic obstructive pulmonary disease; OR: odds ratio

	Binge drinkers	N	Participants with COPD, emphysema, or chronic bronchitis, n (%)	Participants without COPD, emphysema, or chronic bronchitis, n (%)	OR (CI)
Age group, years					
18-24	Yes	6,132	99 (1.6%)	6,033 (98.4%)	1.045 (0.82, 1.322)
	No	17,655	273 (1.5%)	17,382 (98.5%)	
25-44	Yes	23,043	583 (2.5%)	22,460 (97.5%)	0.867 (0.789, 0.952)^*^
	No	70,712	2,055 (2.9%)	68,657 (97.1%)	
45-64	Yes	19,726	1,373 (7%)	18,353 (93%)	0.818 (0.771, 0.868)
	No	110,766	9,278 (8.4%)	101,488 (91.6%)	
65+	Yes	7,804	1,065 (13.6%)	6,739 (86.4%)	1.113 (1.04, 1.19)
	No	136,450	16,971 (12.4%)	119,479 (87.6%)	
Gender					
Male	Yes	34,405	1,806 (5.2%)	32,599 (94.8%)	0.674 (0.64, 0.71)^*^
	No	149,850	11,380 (7.6%)	138,470 (92.4%)	
Female	Yes	22,300	1,314 (5.9%)	20,986 (94.1%)	0.614 (0.579, 0.65)^*^
	No	185,733	17,197 (9.3%)	168,536 (90.7%)	
Race					
White, non-Hispanic	Yes	41,632	2,385 (5.7%)	39,247 (94.3%)	0.599 (0.573, 0.625)^*^
	No	245,300	22,604 (9.2%)	222,696 (90.8%)	
Black, non-Hispanic	Yes	3,272	225 (6.9%)	3,047 (93.1%)	0.868 (0.75, 1.002)
	No	26,324	2,063 (7.8%)	24,261 (92.2%)	
Non-Hispanic/other race	Yes	10,506	424 (4%)	10,082 (96%)	0.71 (0.638, 0.788)^*^
	No	54,446	3,046 (5.6%)	51,400 (94.4%)	

In the 18-24 years age group, the likelihood of COPD, emphysema, or chronic bronchitis was 4.5% lower (OR: 1.045) in binge drinkers, while in the 25-44 years age group, it was 13.3% lower (OR: 0.867). Similarly, in the 45-64 years age group, the likelihood of COPD, emphysema, or chronic bronchitis was 18.2% lower (OR: 0.818) in binge drinkers, and in participants aged 65 years and above, it was 11.3% lower (OR: 1.113). When considering gender, the likelihood of COPD, emphysema, or chronic bronchitis was 32.6% lower (OR: 0.674) in male binge drinkers and 38.6% lower (OR: 0.614) in female binge drinkers compared to their non-binge drinkers’ counterparts. Among racial groups, the likelihood of COPD, emphysema, or chronic bronchitis was 40.1% lower (OR: 0.599) in white binge drinkers, 13.2% lower (OR: 0.868) in black binge drinkers, and 29% lower (OR: 0.71) in non-Hispanic/other race binge drinkers compared to non-binge drinkers. These findings suggest a reduced likelihood of COPD, emphysema, or chronic bronchitis in binge drinkers across all demographic groups.

Table 3 shows the prevalence of COPD, emphysema, or chronic bronchitis in binge drinkers and non-binge drinkers groups based on socioeconomic characteristics. The prevalence of COPD, emphysema, or chronic bronchitis in participants reported as binge drinkers was 1,409 (8.7%) in those with basic education and 1,704 (4.2%) in those with advanced education. When income was considered, COPD, emphysema, or chronic bronchitis was more prevalent in participants with an annual income of <$50,000 (n=1,568, 9.9%) in binge drinkers, compared to a prevalence of 1,138 (3.3%) among participants with an income of >$50,000. The likelihood of an association between COPD, emphysema, or chronic bronchitis and binge drinkers, expressed as OR, was 32.1% lower (OR: 0.679) in participants with basic education and 40.5% lower (OR: 0.595) in those with advanced education. Similarly, the likelihood of association was 30% lower (OR: 0.7) in participants with an annual income of <$50,000 and 30% lower (OR: 0.7) in those with an annual income of >$50,000.

**Table 3:** Prevalence of binge drinking in participants with self-reported COPD, emphysema, or chronic bronchitis based on socioeconomic characteristics ^*^P-value <0.05 was considered statistically significant CI: confidence Interval; COPD: chronic obstructive pulmonary disease; OR: odds ratio

	Binge drinkers	N	Participants with COPD, emphysema, or chronic bronchitis, n (%)	Participants without COPD, emphysema, or chronic bronchitis, n (%)	OR (CI)
Education level					
Basic education	Yes	16,115	1,409 (8.7%)	14,706 (91.3%)	0.679 (0.641, 0.72)^*^
	No	99,640	12,316 (12.4%)	87,324 (87.6%)	
Advanced education	Yes	40,444	1,704 (4.2%)	38,740 (95.8%)	0.595 (0.565, 0.626)^*^
	No	234,698	16,156 (6.9%)	218,542 (93.1%)	
Annual income					
	Yes	15,857	1,568 (9.9%)	14,289 (90.1%)	0.7 (0.662, 0.74)^*^
	No	116,600	15,798 (13.5%)	100,802 (86.5%)	
>$50,000	Yes	34,029	1,138 (3.3%)	32,891 (96.7%)	0.7 (0.656, 0.746)^*^
	No	156,077	7,350 (4.7%)	148,727 (95.3%)	

## Discussion

A retrospective study was conducted using the 2022 BRFSS database to assess the association of COPD with binge drinking in the United States. The dataset included 392,288 participants and analyzed variables related to demographic and socioeconomic characteristics. This study revealed a significant inverse association between binge drinking and COPD. Findings indicated that the likelihood of developing COPD was 5.5% among binge drinkers compared to 8.5% among non-binge drinkers (OR: 0.626; 95% CI: 0.602, 0.65) (Table 1). This aligns with the findings of Yeboah-Kordieh et al. [8], who observed a protective effect of moderate alcohol consumption against COPD, while Greene et al. [9] did not find a significant relationship in a veteran population. Tabak et al. [10] similarly found that moderate alcohol consumption was linked to better pulmonary function and reduced COPD-related mortality. The study also explored demographic variations throughout the data. The prevalence of COPD increased with age among binge drinkers but remained consistently lower compared to non-binge drinkers. White non-Hispanic participants exhibited a stronger inverse association (OR: 0.599; 95% CI: 0.573, 0.625), in line with findings by Kaluza et al. [4], who emphasized the importance of stratification by demographics. Gender differences also showed lower COPD prevalence among male and female binge drinkers. The study also revealed that socioeconomic status moderated the relationship. Higher education and income levels were associated with a stronger inverse association, consistent with Wetherbee et al. [11] and Sisson et al. [12], who noted the influence of lifestyle factors and oxidative stress on lung health. Additionally, Kershaw and Guidot [13] described how chronic alcohol use can damage the lungs’ antioxidant defenses and impair epithelial function. These findings partially align with Ekström et al. [14], who reported that alcohol, particularly heavy consumption, may exacerbate respiratory issues in smokers. While our study found an inverse association, Happel et al. [15] cautioned that chronic alcohol use may lead to immune dysfunction, increasing the risk of respiratory complications. Discrepancies across studies may stem from population differences, measurement methods, and confounding variables such as smoking or comorbidities. In summary, this study offers new insights into the complex relationship between alcohol use and respiratory outcomes, supporting the hypothesis that moderate alcohol intake may have protective effects on lung health. However, further longitudinal studies are essential to confirm causality and clarify mechanisms.

Our study further revealed that socioeconomic characteristics moderated the association between binge drinking and COPD. Participants with basic education levels had an 8.7% prevalence of COPD among binge drinkers vs. 12.4% among non-binge drinkers. Those with advanced education levels showed a lower prevalence, at 4.2% for binge drinkers vs. 6.9% for non-binge drinkers (OR: 0.595; 95% CI: 0.565, 0.626). Similarly, participants with annual incomes below $50,000 had a higher prevalence (9.9% for binge drinkers vs. 13.5% for non-binge drinkers) compared to those earning above $50,000 (3.3% vs. 4.7%). These findings align with Yeboah-Kordieh et al. [8] and highlight the influence of socioeconomic status on respiratory health outcomes. Our findings align partially with prior studies that explored lifestyle risk factors and COPD. Yeboah-Kordieh et al. [8] suggested a protective role of moderate alcohol consumption against COPD, consistent with our observed inverse relationship between binge drinking and COPD. However, Greene et al. did not observe a significant association in a veteran population [9]. These discrepancies may be attributed to differences in study populations, design, and confounding factors like smoking status.

Limitations

This study has some limitations. The reliance on self-reported data for both COPD diagnosis and binge drinking may introduce minor recall bias. While key demographic and socioeconomic variables were considered, unmeasured factors such as genetics or occupational exposures might also have had an influence. Additionally, smaller sample sizes in certain subgroups, particularly related to racial and ethnic minorities, may have reduced the precision of some estimates. Nonetheless, these limitations are typical of large population-based surveys and do not minimize the significance of the overall strength of the findings.

## Conclusions

This study highlighted the inverse association between binge drinking and the development of COPD, emphysema, or chronic bronchitis risk across age groups (18-65+ years) in both female and male genders, as well as various ethnic groups/races and socioeconomic groups. Further prospective studies are needed to validate this association and explore the underlying mechanism.
